# Redefining environmental exposure for disease etiology

**DOI:** 10.1038/s41540-018-0065-0

**Published:** 2018-09-01

**Authors:** Stephen M. Rappaport

**Affiliations:** Program in Environmental Health Sciences, School of Public Health, University of California, Berkeley, CA 94720 USA

## Abstract

Etiological studies of human exposures to environmental factors typically rely on low-throughput methods that target only a few hundred chemicals or mixtures. In this Perspectives article, I outline how environmental exposure can be defined by the blood exposome—the totality of chemicals circulating in blood. The blood exposome consists of chemicals derived from both endogenous and exogenous sources. Endogenous chemicals are represented by the human proteome and metabolome, which establish homeostatic networks of functional molecules. Exogenous chemicals arise from diet, vitamins, drugs, pathogens, microbiota, pollution, and lifestyle factors, and can be measured in blood as subsets of the proteome, metabolome, metals, macromolecular adducts, and foreign DNA and RNA. To conduct ‘exposome-wide association studies’, blood samples should be obtained prospectively from subjects—preferably at critical stages of life—and then analyzed in incident disease cases and matched controls to find discriminating exposures. Results from recent metabolomic investigations of archived blood illustrate our ability to discover potentially causal exposures with current technologies.

## Introduction

The publication of the human genome in 2003 led to speculation^1–3^ that genomic technologies would identify the causes of major chronic diseases, particularly cancer and cardiovascular disease, and would lead to personalized strategies for disease prevention. However, most genome-wide-association studies (GWAS) have not detected large effects of common genetic variants on disease incidence.^4,5^ The small effect sizes identified from single nucleotide polymorphisms detected by GWAS (for example Pharoah et al.^6^ and Dehghan et al.^7^) are consistent with studies of monozygotic twins that point to contributions of entire genotypes toward cancer and cardiovascular disease of 8% and 22%, respectively.^8^ Thus, in weighing the relative influences of heritable genetics and environmental exposures on chronic diseases, the modest effects of heritable genetics suggest that exposures and/or gene–environment interactions (G × E) are major causal factors. Indeed, roughly half of the 50 million global deaths in 2010 were attributed to 18 environmental exposures, led by tobacco smoking, particulate air pollution and indoor smoke, high plasma sodium, and alcohol use.^9^ The clear implication is that epidemiologists seeking unknown causes of chronic diseases should employ a balanced strategy that characterizes both heritable genetics and exposures at high resolution. However, because the human genome project focused exclusively on the genome, it did not motivate the discovery of causal exposures. Indeed, etiological research still focuses on only a few hundred chemicals or mixtures that are quantified by combinations of questionnaires, deterministic models and some measurements.^10^ By continuing to explore such a small universe of exposures, we limit our chances to discover unknown causes of disease.

### Defining exposure via the blood exposome

The conundrum, where scientists use high-throughput genomics to detect the effects of heritable genetics on disease incidence, but rely upon low-technology methods to study the effects of exposures, motivated Christopher Wild to promote the concept of an ‘exposome’—representing the totality of exposures received by an individual during life—for etiologic investigations of cancer.^11^ But unlike the genome, which is largely fixed at birth, the exposome has input from both exogenous and endogenous sources that change throughout life. This calls into question the very nature of ‘exposure’ as a variable in studies of disease etiology. Certainly environmental exposures can be related to levels of pollutants in air, water, and food. But do exposures also include input from nutrients, psycho-social stress, infections, and lifestyle factors? And do perturbations in levels of endogenous molecules, such as sterols and hormones, inflammatory proteins, and metabolites generated by intestinal microbiota constitute exposures? Based on results from the Global Burden of Disease Study,^10^ it is reasonable to speculate that all of these sources generate exposures that can contribute to disease risks. The challenge is to find a suitable avenue for investigating these myriad exposures collectively in etiologic research.

Recognizing that disease processes involve chemicals that alter normal function inside the body, Martyn Smith and I suggested in 2010 that the exposome could be considered as the totality of chemicals that can be measured in blood.^12^ We reasoned that fundamental processes of life rely on chemical communication via circulating molecules from both genetic and environmental sources, and that these chemicals can be interrogated in blood. Thus the ‘blood exposome’ offers an efficient means to integrate exposures from all sources.^13^

**Fig. 1:**
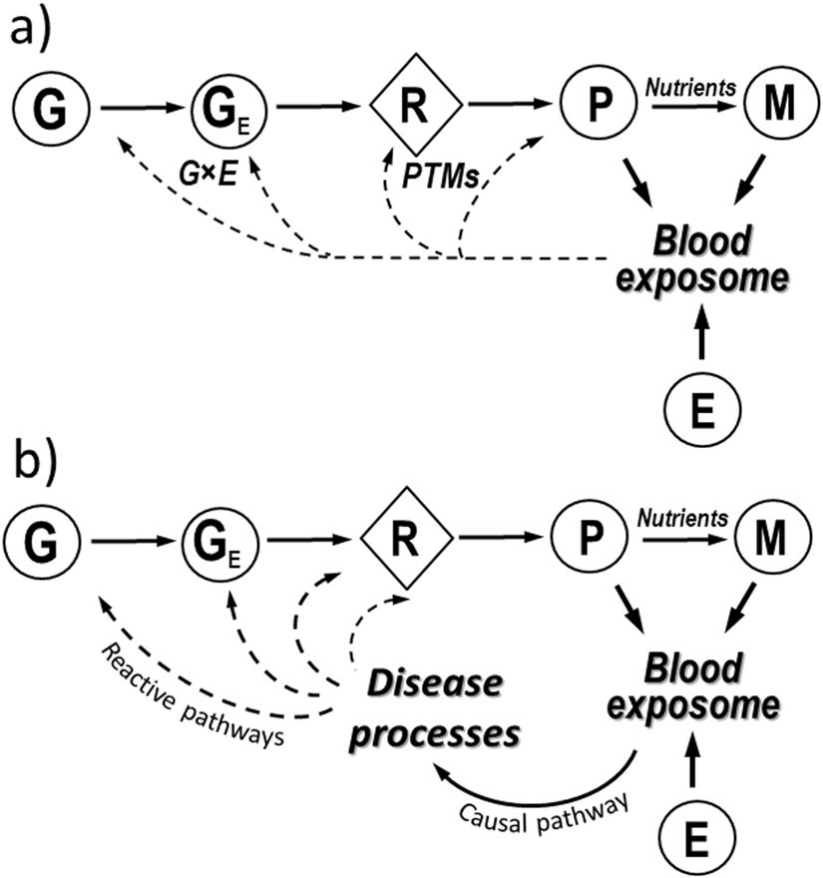
**a** Inputs to the blood exposome from endogenous sources (G, genome; G_E_, epigenome; R, transcriptome; P, proteome; M, metabolome), exogenous exposures (E), post-translational modifications (PTMs) and gene–environment interactions (G × E). **b** Pathways connecting the blood exposome to disease processes (causal pathways) and subsequent feedback to G, G_E_, R, and P (via reactive pathways)

As shown in Fig. 1, endogenous chemicals are generated in the pathway: genome (G), epigenome (G_E_), transcriptome (R), proteome (P), and metabolome (M). The genome interacts with molecules and cells via proteins (for example enzymes, cytokines, receptors, transcription factors, and post-translational modifications) and small molecules (for example amino acids, hormones, lipids, neurotransmitters, human metabolites, and reactive oxygen, and carbonyl species) that are distributed throughout the body by the blood. Indeed, modern medicine relies on surveillance of genome-related factors in blood to evaluate disease risks; for example blood levels of C-reactive protein, fibrinogen, and homocysteine have been used as biomarkers of heart disease for more than a decade.^14^ Careful curation of factors from the genome to the metabolome (Fig. 1) in observational studies can link circulating molecules with genetic loci and reinforces the idea that the proteome and metabolome contribute to the molecular events that underlie disease associations in GWAS.^15–17^

The environmental-exposure component (E) in Fig. 1 represents chemicals from exogenous sources, such as diet, vitamins, drugs, pathogens, microbiota, pollution, and lifestyle factors^12,18^ that can be measured in blood as small molecules,^19^ metals,^20^ antigenic proteins,^21^ and foreign DNA and RNA.^22^ Furthermore, after exogenous chemicals enter the systemic circulation via inhalation, ingestion, or infection, they generate more chemicals via metabolism to reactive intermediates and end products that also enter the blood. Stable adducts of circulating proteins, particularly hemoglobin and human serum albumin, offer avenues for studying the distribution of reactive intermediates that cannot be measured directly in blood.^23^

To glimpse a portion of the blood exposome, Rappaport et al.^13^ examined blood concentrations of 1561 small molecules and inorganic species that had been compiled from healthy individuals (mostly adults) by the National Health and Nutrition Examination Survey (NHANES, www.cdc.gov/nchs/nhanes/index.htm) in samples from the U.S. and the Human Metabolome Database (HMDB, www.hmdb.ca) in samples from throughout the world. These molecules and inorganic species comprised more than 100 chemical classes and displayed an extraordinary 10^11^-fold range of blood concentrations (from fM to mM). Distributions of chemical concentrations derived from food, drugs, and endogenous sources were very similar, whereas blood concentrations of chemicals that were likely results of exposure to pollutants were typically 1000-fold lower than those from the other three sources. Of these 1561 chemicals, 336 had at least one PubMed citation that associated them with a major chronic disease (cardiovascular disease, cancer, or respiratory disease).^13^ Median numbers of PubMed citations per chemical varied significantly across sources of exposure (endogenous, food, drugs, and pollutants) with a typical chemical derived from food being cited about twice as often as one from another source.

### Moving towards exposome-wide-association studies

Untargeted-omics analysis of chemicals in blood samples from patients with disease and healthy controls allows what have been termed exposome-wide-association studies,^24^ which seek to discover discriminating molecular features that may ultimately be linked to causal exposures.^18,24^ Since the proteome, metabolome, and environmental exposures all contribute to the blood exposome (Fig. 1), examples of this type of analysis include proteomics (endogenous and foreign proteins),^25^ metabolomics (small molecules),^19^ metallomics (metals),^20^ adductomics (products of reactive intermediates),^26,27^ and metagenomics (foreign DNA and RNA).^22^ Indeed, it is now feasible to conduct studies that focus on each of these chemical classes separately in human blood or other available biofluids, such as urine or saliva.

The functional genome (genome, epigenome, transcriptome, proteome, and metabolome in Fig. 1) translates genetic information into homeostatic networks of proteins and small molecules. Some of these molecules are causally related to disease processes^28^ (‘causal pathway’ in Fig. 1b). But as a disease progresses, it affects the systems biology in ways that disrupt normal homeostasis, thereby altering the composition of the proteome and metabolome (for example Liddy et al.^29^ and Sekula et al.^30^). These feedback loops, where disease processes alter the functional genome, have been termed ‘reactive pathways’^31^ (Fig. 1b) and can lead to reverse causality in observed associations.^24^ That is, when blood is obtained from disease cases after diagnosis, a protein or small molecule that discriminates between blood samples from cases and controls could have resulted from a reactive pathway rather than a causal pathway. One way to correctly identify the influence of causal environmental exposures is to conduct exposome-wide-association studies with archived blood from disease cases and controls that are nested in prospective cohorts. By using specimens from these cohorts that were collected prior to diagnosis, causal signals are less affected by metabolic dysregulation and the interval between blood collection and diagnosis can be used as a covariate to determine whether a given association is likely to involve reactive pathways.^32^

### Metabolomics-based exposome-wide-association studies

Of the various ‘omics’ methods that can be used to discover environmental exposures associated with disease, metabolomics has received the greatest attention. The current generation of high-resolution liquid chromatography-mass spectrometry (LC-MS) can routinely quantify more than 20,000 small-molecule features in a few microliters of blood,^33^ and online databases facilitate annotation of many analytes.^34^ Nuclear magnetic resonance spectroscopy (NMR) can also be used for untargeted analysis of a much smaller set of abundant small molecules and lipoproteins.^35,36^ When coupled with multivariate analyses to find discriminating small molecules in prediagnostic blood from disease cases and controls, metabonomics^37^ can be regarded as an important subset of exposome-wide-association studies for disease etiology. Table 1 summarizes results from 13 studies that measured small molecules in plasma or serum from incident cases and controls to discover possible causes of cardiovascular disease,^38^ diabetes,^39–43^ and a host of cancers.^44–52^ Periods of follow-up ranged from 2 to 9.6 years and 10 of the 14 studies were performed with LC-MS. Interestingly, only three of the LC-MS studies employed untargeted designs^38,50,51^ and thus many did not take full advantage of the omics capabilities of the analytical platforms. Nonetheless, these studies demonstrate that metabolomics can readily characterize complex mixtures of small molecules in a few microliters of archived blood from incident cases and matched controls. Indeed, most of the studies in Table 1 found significant disease associations with particular molecules. If exposure-related covariates are available from questionnaires or environmental measurements, then a ‘meet-in-the-middle’ strategy can be used to connect discriminating features from untargeted metabolomics with possible exposure sources,^46,53,54^ and such correlations can assist with annotations.

**Table 1:** Recent metabolomics studies that investigated disease associations with small-molecule features in plasma or serum from prospective cohorts

Phenotype	Cohort	Cases/controls	Follow-up (y)	Analytical platform	Design	Exposure variables	Likely associations	Refs.
Cardiovasculardisease	Gene Bank	75/75	≤3	LC-MS	Untargeted	40 Metabolites (out of >2000 detected features) that met ‘acceptance criteria’	18 Small-molecule features of which choline, betaine, and TMAO were annotated	^38^
Type 1 diabetes	DIPP	56/73	3.7	LC-MS	Targeted	53 Lipids	Children who progressed to T1D were deficient in triglycerides and phosphatidyl cholines (possible choline deficiency)	^39^
Type 2 diabetes	FHS	189/189	12	LC-MS	Targeted	61 Polar metabolites and>100 Lipids	Branched-chain and aromatic amino acids increased risk; sets of lipids increased or decreased risk depending on chain length and double bonds	^40, 41^
Type 2 diabetes	SCHS	197/197	6	LC-MS and GC-MS	Untargeted	4859 Polar and nonpolar metabolites	35 Significant associations including branched-chain amino acids & nonesterified fatty acids and lysophosphatadylinositols	^43^
Pre-diabetes	KORA	IFG:102/866, IGT:238/866	7	LC-MS	Targeted	140 Lipids, amino acids, and biogenic amines	26 Associations, with glycine, LPC (18:2), and acetylcarnitine being the strongest	^42^
Gastric cancer	EPIC	238/626	3.2	GC-MS	Targeted	22 Phospholipid fatty acids	Oleic acid, a-linolenic acid, and di-homo-g-linolenic acid	^44^
Breast cancer	EPIC	363/702	7	GC-MS	Targeted	22 Phospholipid fatty acids	Trans-palmitoleic and elaidic acids	^45^
Hepatocellular carcinoma	EPIC	114/122	>2	NMR	Untargeted	8500 NMR bins reduced to 285 clusters of variables	Clusters of sugars, amino acids, lipids and nutrients	^46^
Colorectal cancer	WHI-OS	835/835	5.2	LC-MS	Targeted	Choline and its metabolites	TMAO and betaine/choline ratio	^47^
Colorectal cancer	EPIC	1367/2323	3.7	LC-MS	Targeted	Methionine, choline, betaine and dimethylglycine	Weak associations with methionine, choline and betaine	^48^
Colorectal cancer	PLCO	254/254	7.8	LC-MS and GC-MS	Untargeted	268 Annotated metabolites detected in >80% of specimens	Glycochenodeoxycholate in women but not men	^50^
Pancreatic cancer	HPFS, NHS, PHS, WHI-OS	454/908	8.7	LC-MS	Targeted	83 Polar metabolites	Branched-chain amino acids	^49^
Prostate cancer	ATBC	200/200	≤20	LC-MS & GC-MS	Untargeted	626 Annotated metabolites detected in >95% of specimens	None after Bonferroni correction	^51^
Hepatobiliary cancers	EPIC	HCC:147/147, IHBC:43/43, GBTC:134/134	9.6	LC-MS	Targeted	28 Amino acids, biogenic amines, and total hexoses	HCC: 14 Molecules, mainly branched-chain and aromatic amino acids	^52^

*ATBC* alpha-tocopherol beta-carotene cancer prevention study; *DIPP* Type-1 diabetes prediction and prevention study (birth cohort), *EPIC* European Prospective Investigation into Cancer, *FHS* Framingham Health Study, *GBTC* gallbladder and biliary tract cancers, *GC-MS* gas chromatography-mass spectrometry, *HCC* hepatocellular carcinoma, *HPFS* Health Professionals Follow-up Study, *IFG* impaired fasting glucose, *IGT* impaired glucose tolerance, *IHBC* intrahepatic bile duct cancer, *GBTC* gall-bladder and biliary-tract cancers, *KORA* Cooperative Health Research in the Region of Augsburg cohort, *LC-MS* liquid chromatography-mass spectrometry, *LPC* lysophosphatidylcholine, *NHS* Nurses’ Health Study, *NMR* nuclear mass resonance spectroscopy, *PHS* Physicians’ Health Study, *PLCO* prostate, lung, colorectal, and ovarian cancer screening trial, *SCHS* Singapore Chinese Health Study, *TMAO* trimethylamine-*N*-oxide, *WHI-OS* Women’s Health Initiative-Observational Study

Although the literature summarized in Table 1 is dominated by targeted designs, hypothesis-free exposome-wide-association studies can be performed with untargeted analyses that focus on those features, whose signatures (for example LC-MS peaks defined by accurate molecular mass and chromatographic retention time) differ in abundance between cases and controls.^33^ After highly associated features from this analysis have been identified, the molecules can be targeted in follow-up studies to identify environmental sources or reactive pathways, to establish exposure–response relationships and other evidence supporting causality,^24^ and to direct interventions and predictive modeling. These follow-up studies can employ high-throughput methods to quantify selected analytes in thousands of biospecimens using, for example, triple-quadrupole LC-MS^38,55,56^ or NMR.^35,36^

The untargeted exposome-wide association study conducted by Wang et al.^38^ (Table 1) is noteworthy because the authors found 18 features (out of more than 2000 detected by LC-MS) that were associated with cardiac events in plasma samples from only 75 incident cases and matched controls. Three highly discriminating features were choline (a nutrient) and its metabolites, betaine, and trimethylamine-*N*-oxide (TMAO). As TMAO is a product of joint microbial and human metabolism of choline and carnitine (another nutrient),^55,56^ the positive association between plasma TMAO and cardiac events points to the involvement of dietary factors combined with the gut microbiota in the etiology of cardiovascular disease. Indeed, the early associations detected between plasma TMAO and cardiac events^38^ spawned an extensive set of follow-up studies that employed targeted methods to replicate the findings and to explore contributions of TMAO and the gut microbiota towards development of cardiovascular disease.^57^ It is also interesting that the study by Bae et al.^47^ (Table 1) found a positive association between plasma TMAO and colorectal-cancer incidence, again suggesting involvement of the gut microbiota.

### Time-varying exposures

The blood exposome is dynamic with concentrations of chemicals varying throughout life due to changes in location, physiology, diet, lifestyle, and other factors.^42^ Given the impact of cumulative exposures (‘exposure memory’^58^) on chronic diseases, it is important that exposure monitoring begin in early life. Birth cohorts provide a perfect avenue for obtaining repeated measurements of the blood exposome—beginning at birth and continuing through critical stages of life—that can be used to detect disease associations and windows of susceptibility (for example Oresic et al.^39^). However, any cohort with repeated collection of blood can provide critical information regarding the timing of disease progression (for example Soininen et al.^35^). Neonatal blood spots that are collected at birth to screen for congenital errors in metabolism could be archived for subsequent exposome-wide-association studies to find effects of in utero environmental exposures on pediatric (or later) diseases.^59^

Temporal variability of individuals’ exposomes leads to exposure-measurement errors that attenuate case-control comparisons of blood levels^60^ and thereby reduce the power to detect disease associations. The magnitudes of exposure-measurement errors depend, in part, on the residence times of omics features in the body. Small molecules, which tend to have residence times of less than one day, can have much greater measurement error than longer-lived biomarkers, such as adducts of human serum albumin or hemoglobin which reside in the body for 1–2 months.^60^ However, other factors influence temporal variability in blood concentrations; for example, levels of small molecules under homeostatic control can be quite stable over time.^61^ Cohorts with repeated collection of blood permit cumulative exposures of omics features to be estimated with concomitant reduction in exposure-measurement errors.^60,61^ Although exposure-measurement errors tend to bias case-control comparisons towards the null and thus result in false negatives, associations detected in exposome-wide association studies with single biospecimens from each subject are unlikely to be false positives after adjustment for multiple testing and should be followed up with validation samples.

## Conclusions

Transformative research generally happens once in a generation. Over the last quarter of a century, epidemiologists have emphasized genetic factors as the putative causes of chronic diseases. Because the human genome project planted the seeds for genome sequencing and large-scale GWAS, it was inevitable that these methods would be used to search for disease causes and, in fact, almost 2000 GWAS have been reported.^62^ Yet, virtually all disease-associated variants individually contributed very small risks.^63^ This outcome should not be taken to mean that the totality of genetic risks is trivial. After all, studies of monozygotic twins in Western Europe point to attributable genetic risks of about 8% overall for cancer and 22% for coronary heart disease and accounted for around 250,000 deaths in the year 2000.^8^ Nor do I discount the possibility that genes mainly exert their influence through gene–environment interactions, including epigenetics.^64,65^ But, based on current evidence, there can be little doubt that the next generation of etiological research should move towards environmental exposures as causes of chronic diseases, possibly operating in tandem with genetic factors.

In the age of GWAS it is difficult to reconcile the crude state of knowledge about environmental exposures that has been gleaned from traditional methods.^66^ Indeed, a compelling reason for embracing the blood exposome is the potential to perform exposome-wide-association studies that comprehensively characterize environmental exposures with biospecimens from nested case-control studies or from surveillance of individuals’ blood exposomes via routine screening.^33^ By heightening awareness of the enormous diversity of environmental exposures, the blood exposome should promote the coalescing of etiological research that has been fractured along lines related to exposure sources, for example air, water, diet, microbiota, infections, and psychosocial stress.^12^ To reach their full potential, applications employing human blood or other biofluids for exposome-wide-association studies will require standardization of methods and rigorous multi-step replication in order to find unknown causes of chronic diseases.
